# The Ins and Outs of Aurora B Inner Centromere Localization

**DOI:** 10.3389/fcell.2017.00112

**Published:** 2017-12-22

**Authors:** Sanne Hindriksen, Susanne M. A. Lens, Michael A. Hadders

**Affiliations:** Oncode Institute, Center for Molecular Medicine, University Medical Center Utrecht, Utrecht University, Utrecht, Netherlands

**Keywords:** Aurora B, Chromosomal Passenger Complex, Haspin, Bub1, Shugoshin, chromosome segregation, centromere, mitosis

## Abstract

Error-free chromosome segregation is essential for the maintenance of genomic integrity during cell division. Aurora B, the enzymatic subunit of the Chromosomal Passenger Complex (CPC), plays a crucial role in this process. In early mitosis Aurora B localizes predominantly to the inner centromere, a specialized region of chromatin that lies at the crossroads between the inter-kinetochore and inter-sister chromatid axes. Two evolutionarily conserved histone kinases, Haspin and Bub1, control the positioning of the CPC at the inner centromere and this location is thought to be crucial for the CPC to function. However, recent studies sketch a subtler picture, in which not all functions of the CPC require strict confinement to the inner centromere. In this review we discuss the molecular pathways that direct Aurora B to the inner centromere and deliberate if and why this specific localization is important for Aurora B function.

## Introduction

**Figure 1:**
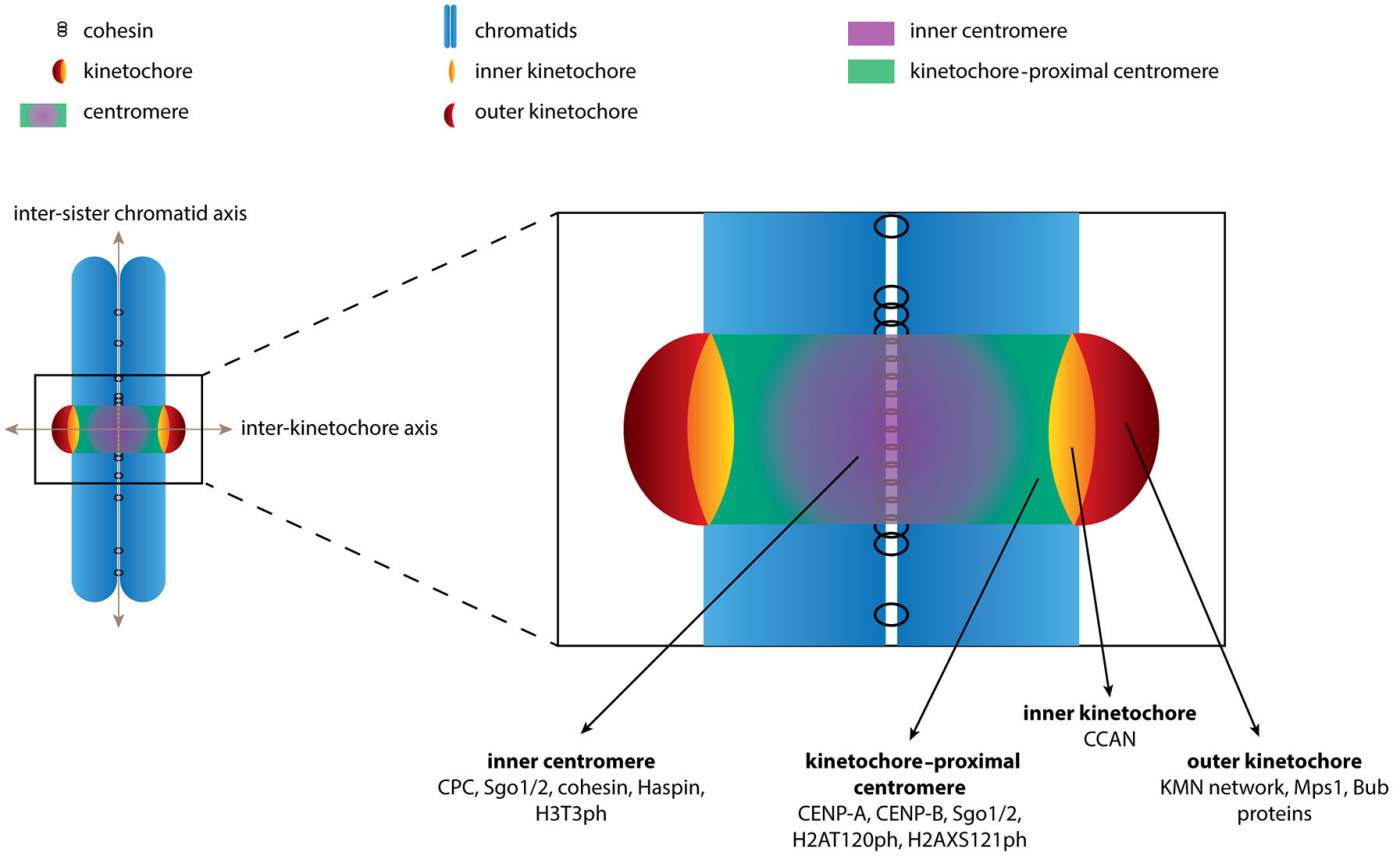
Schematic depiction of the chromosome regions described in this review. The boundaries of various chromosome regions are shown. Examples of (phospho)proteins and protein complexes that localize to each of the regions are indicated. For sake of clarity we have limited the number of proteins depicted and by no means is the list intended to be comprehensive.

The segregation of chromosomes during mitosis is perhaps the most dramatic event of the cell cycle. The underlying forces that mediate the division of our DNA must be tightly controlled to ensure accurate distribution of the genomic content between the newly formed daughter cells. The mitotic kinase Aurora B plays a crucial role in this process. Aurora B is the enzymatic component of a larger protein assembly, termed the Chromosomal Passenger Complex (CPC). In mitosis, the CPC is concentrated at the inner centromere, a highly specialized region on the chromatin where the inter-sister chromatid axis and the inter-kinetochore axis intersect (Figure 1). Concentrating the CPC at the centromere region is considered crucial for its function as it places Aurora B in close proximity to its substrates on kinetochores. At the same time, its confinement to the inner centromere is thought to allow discrimination between spatially separated substrates (Liu et al., 2009; Welburn et al., 2010). Multiple regulatory networks involving multiple mitotic kinases determine the site of CPC activity. Intriguingly, these networks are themselves centered on Aurora B activity and thus involve multiple feedback circuits. In this review, we discuss the molecular determinants that guide the CPC toward the (inner) centromere and deliberate on the relevance of this specific localization for Aurora B function.

## The chromosomal passenger complex

In addition to Aurora B, the CPC consists of the accessory subunits Borealin, Survivin, and INCENP. The complex can be divided into two functional modules that are bridged by INCENP. The first module consists of Borealin and Survivin, which bind to the N-terminal region of INCENP (referred to as the CEN module) and together control the localization of the CPC (Klein et al., 2006; Jeyaprakash et al., 2007; Carmena et al., 2012). The second module harbors the activity of the CPC and consists of Aurora B associated with a region at the C-terminus of INCENP (known as the IN-box). The interaction between Aurora B and the IN-box not only links Aurora B to the CPC but is also required for its full kinase activity (Bishop and Schumacher, 2002; Honda et al., 2003; Sessa et al., 2005).

The CPC is first observed in the nucleus in late S phase with expression peaking in G2/M phase (Carmena et al., 2012). Upon entry into mitosis all CPC subunits can be observed along the length of the chromosome arms although their localization quickly becomes restricted to centromeres, specifically at the inter-sister chromatid region referred to as the inner centromere (Figure 1). At anaphase onset the CPC translocates to the central spindle where Aurora B activity contributes to cytokinesis (Carmena et al., 2012). In this review, however, we will only focus on the role of the CPC at the inner centromere.

We stress the use of consistent terminology when describing the various pools of proteins at the centromere region. First, we define the inner centromere as the intersection of the inter-sister chromatid axis and the inter-kinetochore axis (Figure 1). Proteins found at the inner centromere include the cohesin complex, the CPC, Shugoshin 1/2 (Sgo1/2), Haspin and the phospho-mark H3T3ph, which are typically observed as a single focus in between the kinetochores (Figure 1). Second, we define the region located more outward along the inter-kinetochore axis as the kinetochore-proximal centromere. Proteins found at the kinetochore-proximal centromere include CENP-A, CENP-B, Sgo1/2 and the phospho-mark H2AT120ph, which are observed as two foci adjacent to kinetochores (Figure 1). The final region along the inter-kinetochore axis consists of the kinetochores, large protein structures assembled on CENP-A containing chromatin. The kinetochore can further be divided into the inner kinetochore, consisting of the CCAN (Constitutively Centromere Associated Network) proteins and the outer kinetochore, which includes the KMN network (comprising Knl1, the Mis12 complex and the NDC80 complex), Bub1 and Mps1. These proteins are also observed as two foci located further apart along the inter- kinetochore axis (Figure 1). Confusion arises as proteins are often labeled as belonging to “a kinetochore pool” based solely on the observation of two distinct foci. While it may be difficult to discriminate between the kinetochore-proximal centromere and the kinetochores experimentally we believe the distinction is important when discussing the spatial regulation of CPC function.

## How is inner centromere localization of the CPC regulated?

### The two recruitment arms that control inner centromere localization of the CPC

**Figure 2:**
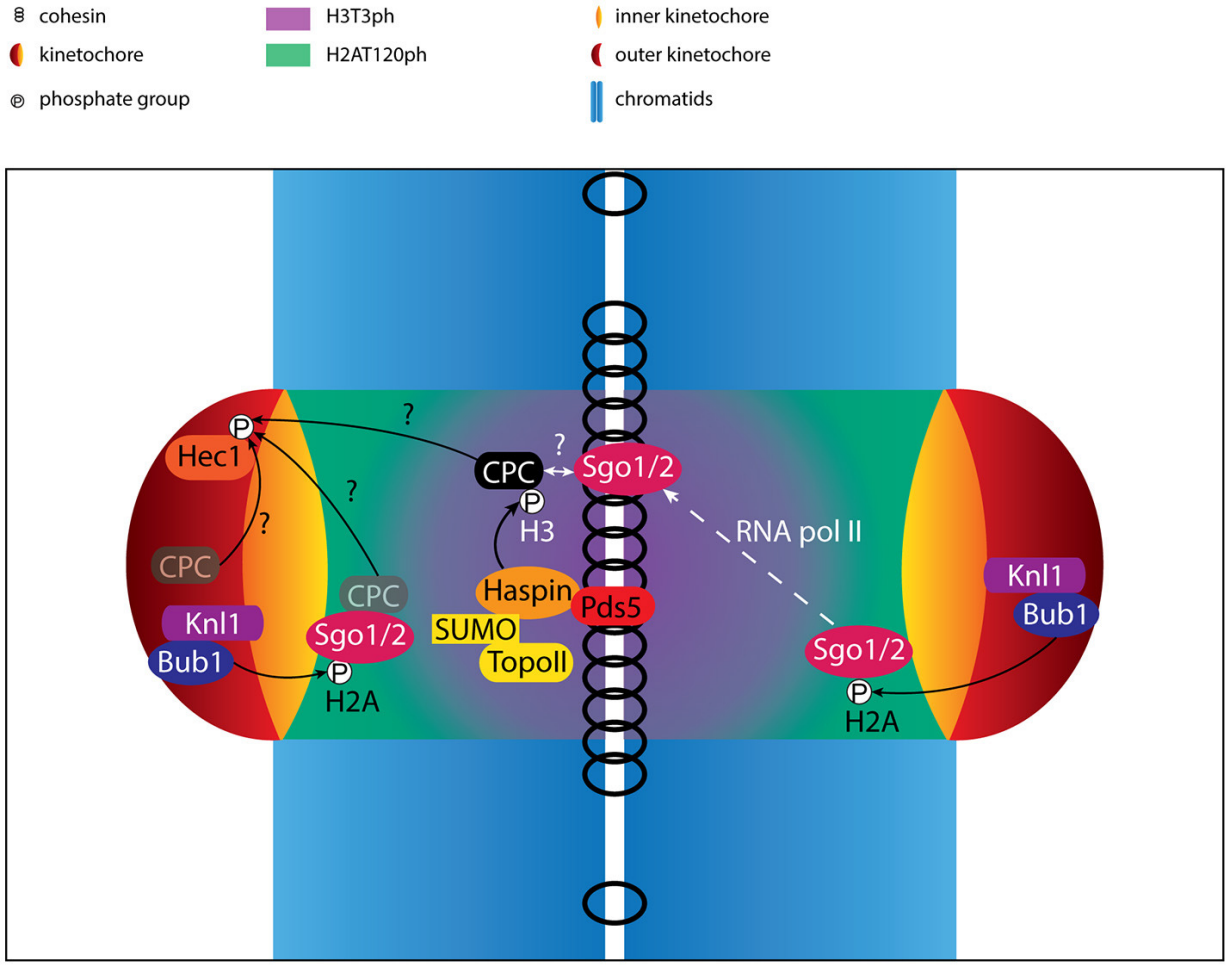
Regulation of centromere/kinetochore proteins in (pro)metaphase. Cartoon depicting the localization and interactions of centromere- and kinetochore proteins. Phosphorylation events are indicated. The activity of RNA polymerase II (RNA pol II) contributes to the translocation of Sgo1/2 from the kinetochore-proximal centromere to the inner centromere. Besides the well-established pool of CPC at the inner centromere, putative pools of CPC at the kinetochore and kinetochore-proximal centromere are shown (opaque). The CPC pool that mediates phosphorylation of outer kinetochore substrates such as Hec1 remains unclear, and is indicated by question marks. Finally, although the CPC has been shown to interact with Sgo1/2, it is uncertain how and where this interaction takes place and how this interaction contributes to inner centromere localization of the CPC, also depicted by a question mark.

Inner centromere localization of the CPC depends on the activity of two histone kinases, Haspin and Bub1, which phosphorylate histone H3 on threonine 3 (H3T3ph) and histone H2A on threonine 120 (H2AT120ph) respectively. These phospho-marks are believed to function as the centromeric receptors for the CPC (Figure 2). The CPC can indeed bind directly to H3T3ph via Survivin and depletion of Haspin or inhibition of Haspin kinase activity results in dispersion of the CPC over the chromosome arms (Kelly et al., 2010; Wang et al., 2010, 2012; Yamagishi et al., 2010; De Antoni et al., 2012). Moreover, mutations in the BIR domain of Survivin that abrogate H3T3ph binding result in a similar phenotype (Lens et al., 2006; Yue et al., 2008; Kelly et al., 2010; Wang et al., 2010; Yamagishi et al., 2010). However, while the CPC becomes largely dispersed over the chromatin in the absence of Haspin activity, a residual centromeric pool of the CPC adjacent to kinetochores has been observed (Bekier et al., 2015). As of yet it is unclear what controls the localization of the CPC in the absence of Haspin activity, however, it is tempting to speculate the involvement of Bub1 and Sgo1/2, as the CPC appears to co-localize with H2AT120ph at the kinetochore-proximal centromeres in this case (Bekier et al., 2015). Alternatively, the two foci may represent a kinetochore pool of the CPC, as previously suggested by DeLuca *et al*. based on their observation of an active pool of Aurora B at the kinetochores, using phospho-specific antibodies (DeLuca et al., 2011; Caldas et al., 2013).

**Figure 3:**
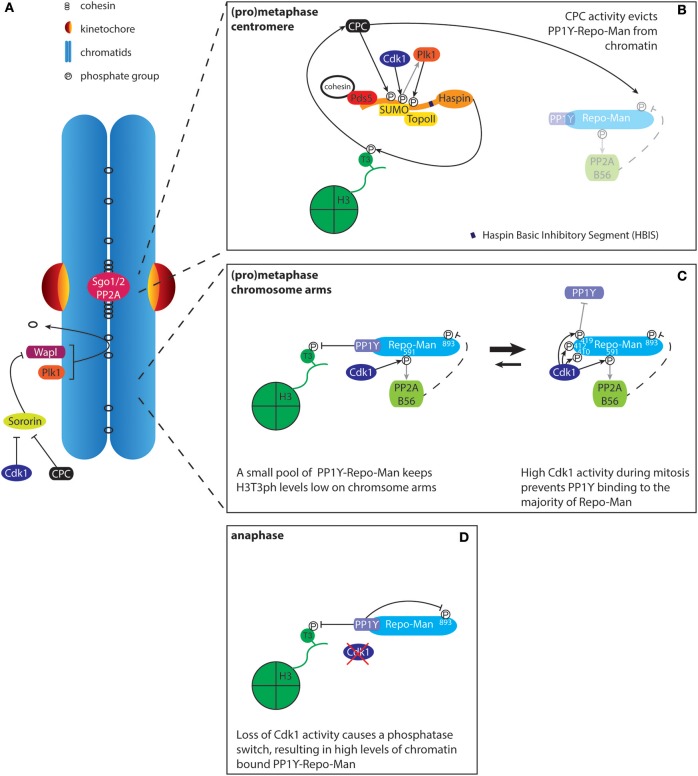
Regulation of histone H3T3 phosphorylation by Haspin and PP1Y-Repo-Man. **(A)** The prophase pathway removes cohesin from the chromosome arms. Sororin binds to the cohesin complex and this interaction is required for maintaining stable cohesion. During mitosis, Plk1 phosphorylates the cohesin subunit SA2 while Cdk1 and Aurora B phosphorylate Sororin. This results in the release of Sororin from cohesin, leading to the Wapl dependent removal of cohesin from chromatin. Centromeres are protected against the prophase pathway through recruitment of Sgo1/2-PP2A. The recruitment of Sgo1-PP2A results in de-phosphorylation of SA2 and Sororin, rendering the centromeric cohesin complexes resistant to Wapl activity. Effectively, this results in the concentration of cohesin/Pds5A/B and thus Haspin at centromeres, thereby contributing to the defined localization of the CPC at the inner centromere. **(B)** The cohesin-associated protein Pds5A/B, in conjunction with SUMOylated Topoisomerase II (TopoII), recruits Haspin to the inner centromere. Haspin phosphorylation by Aurora B (CPC), Cdk1, and Plk1 releases HBIS dependent Haspin auto-inhibition. Phosphatase activity toward H3T3ph by PP1Y-Repo-Man is inhibited through phosphorylation of Repo-Man by the CPC, which prevents Repo-Man recruitment to chromatin. **(C)** At the chromosome arms, Haspin levels are lower, most likely due to reduced levels of cohesin and SUMOylated TopoII. Low levels of chromatin targeted PP1Y-Repo-Man are sufficient to maintain H3T3 in a dephosphorylated state. **(D)** Upon anaphase onset, loss of Cdk1 activity promotes the PP1Y-Repo-Man interaction, resulting in high levels of the active complex associated with chromatin.

The interaction between H2AT120ph and the CPC requires the Shugoshin paralogs, Sgo1 and Sgo2. Sgo1 and Sgo2 localize to centromeres in a Bub1 dependent fashion, where they play a crucial role in protecting centromeric cohesin from Wapl- and Plk1-dependent removal during prophase (Figure 3A) (Salic et al., 2004; Tang et al., 2004; Kitajima et al., 2005, 2006; McGuinness et al., 2005; Gandhi et al., 2006; Kueng et al., 2006; Tanno et al., 2010; Haarhuis et al., 2013; Tedeschi et al., 2013). Sgo1 and Sgo2 directly interact with both H2AT120ph and the CPC, suggesting they may serve as adaptors that control centromere localization of the CPC (Kawashima et al., 2007, 2010; Tsukahara et al., 2010; Yamagishi et al., 2010; Liu et al., 2015; Baron et al., 2016). Indeed, depletion of Sgo1 and Sgo2 results in a marked decrease in centromere levels of the CPC, similar to what is observed for the depletion or inhibition of Bub1 (Yamagishi et al., 2010; Baron et al., 2016).

It is clear that Haspin and Bub1, in conjunction with Sgo1 and Sgo2, cooperate to define a unique chromatin environment that supports recruitment of the CPC toward the inner centromere. Major advances in the past decade have resulted in a picture that suggests that Haspin and Bub1 exert their control over the CPC by regulating two distinct axes along mitotic chromosomes: while Haspin-H3T3ph facilitates recruitment of the CPC toward the inter-sister chromatid axis the Bub1-H2AT120ph-Sgo1/2 pathway restricts the inter-sister chromatid pool of the CPC to centromeres. Together, these pathways form an evolutionarily conserved mechanism that defines the inner centromere (Table 1) (Yamagishi et al., 2010).

**Table 1:** Conservation of Aurora B, INCENP, Borealin, Survivin, Bub1, Sgo1, Sgo2, and Haspin among species.

**Species**	**Protein**	**References**	**Protein**	**References**	**Protein**	**References**	**Protein**	**References**
*H. sapiens*	Aurora B/AIM1	Bischoff et al., 1998; Kimura et al., 1998; Shindo et al., 1998; Tatsuka et al., 1998	INCENP	Mackay et al., 1993; Eckley et al., 1997; Adams et al., 2001a	Survivin	Ambrosini et al., 1997; Li et al., 1998; Skoufias et al., 2000; Uren et al., 2000	Borealin	Gassmann et al., 2004; Chang et al., 2006
*M. musculus*	Aurora B/AIM1	Niwa et al., 1996; Shindo et al., 1998	INCENP	Fowler et al., 1998; Saffery et al., 1999	Survivin	Li and Altieri, 1999; Uren et al., 2000	Borealin	Gassmann et al., 2004; Yamanaka et al., 2008; Zhang et al., 2009
*X. laevis*	xAurora B/AIRK2	Adams et al., 2000	xINCENP	Stukenberg et al., 1997; Adams et al., 2000	xSurvivin	Bolton et al., 2002; Murphy et al., 2002	Dasra-A, Dasra-B	Gassmann et al., 2004; Sampath et al., 2004
*D. melanogaster*	ial	Reich et al., 1999; Giet and Glover, 2001	Incenp	Adams et al., 2000, 2001b	Deterin/dSurvivin	Jones et al., 2000; Szafer-Glusman et al., 2011	Borr	Gassmann et al., 2004; Sampath et al., 2004; Hanson et al., 2005
*C. elegans*	AIR-2	Schumacher et al., 1998	ICP-1	Adams et al., 2000; Kaitna et al., 2000	BIR-1	Uren et al., 1998; Fraser et al., 1999; Speliotes et al., 2000	CSC-1	Romano et al., 2003; Gassmann et al., 2004
*S. cerevisae*	Ipl1	Chan and Botstein, 1993; Francisco et al., 1994	Sli15	Kim et al., 1999; Adams et al., 2000; Kang et al., 2001	Bir1	Uren et al., 1998, 1999; Yoon and Carbon, 1999; Li et al., 2000	Nbl1	Nakajima et al., 2009
*S. pombe*	Ark1	Petersen et al., 2001	Pic1	Adams et al., 2000; Leverson et al., 2002	Bir1/Cut17/Pbh1	Uren et al., 1998, 1999; Rajagopalan and Balasubramanian, 1999; Morishita et al., 2001	Nbl1	Bohnert et al., 2009
*H. sapiens*	Bub1	Pangilinan et al., 1997; Cahill et al., 1998; Ouyang et al., 1998; Boyarchuk et al., 2007; Kawashima et al., 2010; Yamagishi et al., 2010	Sgo1	Salic et al., 2004; Kitajima et al., 2005; McGuinness et al., 2005; Kawashima et al., 2007; Tsukahara et al., 2010	Sgo2	Kitajima et al., 2006; Huang et al., 2007; Tsukahara et al., 2010	Haspin	Higgins, 2001b; Tanaka et al., 2001; Dai and Higgins, 2005; Wang et al., 2010; Yamagishi et al., 2010
*M. musculus*	Bub1	Pangilinan et al., 1997; Taylor et al., 1998; Ricke et al., 2012	Sgo1	Salic et al., 2004; McGuinness et al., 2005; Lee et al., 2008	Sgo2	Lee et al., 2008; Llano et al., 2008	Haspin	Tanaka et al., 1999; Nguyen et al., 2014; Shimada et al., 2016
*X. laevis*	xBub1	Schwab et al., 2001; Sharp-Baker and Chen, 2001; Boyarchuk et al., 2007; Williams et al., 2017	xSgo1	Salic et al., 2004; Rivera and Losada, 2009	xSgo2	Rivera et al., 2012	xHaspin	Kelly et al., 2010; Ghenoiu et al., 2013; Yoshida et al., 2016
*D. melanogaster*	Bub1	Basu et al., 1999	MEI-S332	Goldstein, 1980; Kerrebrock et al., 1992; Moore et al., 1998; Resnick et al., 2006	(only one Sgo identified)		Haspin	Higgins, 2001a; Xie et al., 2015
*C. elegans*	BUB-1	Pangilinan et al., 1997; Oegema et al., 2001; Moyle et al., 2014	SGO-1 (may not be required for AIR-2 regulation)	Rabitsch et al., 2004; de Carvalho et al., 2008	(only one Sgo identified)		C01H6.9 (hasp-1); Y18H1A.10 (hasp-2); F22H10.5; W02H3.2; Y40A1A.1 (there may be up to 15 Haspin paralogs)	Higgins, 2001a, 2003; Cuppen et al., 2003
*S. cerevisae*	Bub1	Hoyt et al., 1991; Roberts et al., 1994; Fernius and Hardwick, 2007	Sgo1	Kitajima et al., 2004; Indjeian et al., 2005; Riedel et al., 2006; Fernius and Hardwick, 2007; Kiburz et al., 2008; Peplowska et al., 2014; Edgerton et al., 2016	(only one Sgo identified)		Alk1/Alk2	Higgins, 2001a; Nespoli et al., 2006; Edgerton et al., 2016
*S. pombe*	Bub1	Bernard et al., 1998; Kawashima et al., 2010	Sgo1 (important for cohesion maintenance in meiosis)	Kitajima et al., 2004; Rabitsch et al., 2004; Riedel et al., 2006; Kawashima et al., 2010	Sgo2 (important for CPC recruitment, interacts with Bir1)	Kitajima et al., 2004; Rabitsch et al., 2004; Kawashima et al., 2007, 2010; Vanoosthuyse et al., 2007; Tsukahara et al., 2010	Hrk1	Higgins, 2001a; Yamagishi et al., 2010

*The references for each protein are listed in the column to the right of the corresponding protein. The references were selected based on their description of identification of the gene that encodes the indicated protein, characterization of the protein, and/or insight into the regulation of/by the protein*.

### Haspin, H3T3ph and the inter-sister chromatid axis

Clearly, the mitotic kinase Haspin plays a crucial role in regulating CPC localization (Kelly et al., 2010; Wang et al., 2010). By phosphorylating H3T3 it generates a receptor that directly recruits the CPC via Survivin. This raises the question how H3T3 phosphorylation becomes enriched at the inner centromere. The data so far point to a complex regulatory network that involves the regulation of Haspin localization and kinase activity, the spatial control of the H3T3ph counteracting phosphatase Protein Phosphatase 1γ (PP1γ), and perhaps the presence of other epigenetic marks within the H3 tail that changes its ability to serve as a substrate for Haspin.

#### Regulation of Haspin localization

Defining the native localization of Haspin has been hampered by technical challenges. Available antibodies do not allow detection of endogenous Haspin by immunofluorescence, suggesting that the protein is likely expressed at very low levels (Higgins, 2001b). Indeed, endogenous Haspin tagged with YFP is observed at very low levels on mitotic chromatin (Hindriksen et al., 2017). This localization pattern appears similar to what has been observed for overexpressed GFP-Haspin (Dai et al., 2005). More detailed analysis of chromosome spreads has revealed that ectopically expressed GFP-Haspin is concentrated at the inner centromere, coinciding with H3T3ph and the CPC (Dai et al., 2005; Yamagishi et al., 2010; Yoshida et al., 2016; Goto et al., 2017). Multiple factors contribute to the defined localization of Haspin at the inner centromere. First, Haspin directly interacts with the cohesin-associated proteins Pds5A/B (Yamagishi et al., 2010; Carretero et al., 2013; Goto et al., 2017; Zhou et al., 2017). The cohesin complex is established along the entire length of the inter-sister chromatid axis during S phase (Figure 1). However, the majority is removed early in mitosis. This process is termed the prophase pathway and depends on the activity of Wapl and Plk1 (Figure 3A) (Hauf et al., 2005; Haarhuis et al., 2014). Centromeres are resistant to the cohesin removing activity of Wapl and Plk1 due to the presence of Sgo1 that is bound to the phosphatase PP2A, thereby keeping sister chromatids together until anaphase onset (Haarhuis et al., 2014). Thus, through the progressive removal of cohesin complexes from the chromosome arms, the prophase pathway is thought to contribute to the concentration of the cohesin complex, including Pds5A/B and Haspin, at the inner centromere (Watanabe, 2010). In line with this model, interfering with the prophase pathway, through depletion of Wapl, results in a more dispersed localization of the CPC along the chromosome arms (Haarhuis et al., 2013; Tedeschi et al., 2013). Interestingly, recent work has demonstrated that Haspin and Wapl compete for the same binding site on Pds5B. Therefore, Haspin also directly contributes to centromeric cohesion protection (Goto et al., 2017; Zhou et al., 2017).

In addition, recent work has highlighted a role for Topoisomerase II (TopoII) in Haspin recruitment. TopoII plays a central role in the architecture of mitotic chromatin but also resolves topological problems, for example those that arise during DNA replication (Nitiss, 2009). TopoII displays a distinct axial localization along the chromosomes, and also accumulates at centromeres where its activity resolves topologically linked sister chromatids, or catenanes, prior to and during early anaphase (Earnshaw and Heck, 1985; Rattner et al., 1996; Christensen et al., 2002; Tavormina et al., 2002; Hudson et al., 2003; Kireeva et al., 2004; Lee and Bachant, 2009; Samejima et al., 2012; Hengeveld et al., 2015). Depletion of TopoII in *Drosophila melanogaster* (*Dm*) S2 cells was shown to result in delocalization of the CPC to kinetochore-proximal centromeres, reminiscent of the residual pool of the CPC observed upon Haspin inhibition in human cells (Coelho et al., 2008; Bekier et al., 2015). In line with this, recent work in yeast and frogs has demonstrated that the altered localization of the CPC upon TopoII depletion is likely due to disruption of Haspin recruitment to chromatin. Recruitment of Haspin does not require TopoII catalytic activity but instead depends on the modification of the TopoII C-terminal domain (CTD) with a Small Ubiquitin-like MOdifier (SUMO) (Edgerton et al., 2016; Yoshida et al., 2016; Goto et al., 2017). SUMOylation of TopoII is required for TopoII enrichment at centromeres during mitosis (Azuma et al., 2003, 2005; Díaz-Martínez et al., 2006; Dawlaty et al., 2008). Haspin contains a SUMO interacting motif (SIM) that binds to SUMOylated TopoII. Additionally, this interaction strongly depends on phosphorylation of Haspin by Cdk1. It is unclear if TopoII also contributes to Haspin recruitment in human cells. However, the presence of a SIM is conserved in human Haspin and SUMOylation is high at centromeres during mitosis (Zhang et al., 2008).

So far the data indicate that cohesin-Pds5A/B and TopoII-SUMO collaborate to control the localization of Haspin to the inner centromere. This suggests that coincidence detection, the requirement for simultaneous binding of Haspin to Pds5A/B and SUMO-conjugated TopoII, may serve to restrict Haspin localization to the inner centromere. At the same time the prophase pathway likely contributes to this process through the removal of cohesin from the chromosome arms.

While these data provide an explanation for the observed enrichment of Haspin around the centromeres during mitosis, it should be noted that both in and out of mitosis most of Haspin is associated with chromatin along the entire chromosomal arms, where the levels of cohesin and SUMO conjugated TopoII are low. This argues that alternative factors may further contribute to chromatin association of Haspin. Alternatively, chromatin association of Pds5A/B may be differentially regulated from cohesin. Pds5A/B do not belong to the core components of the cohesin complex and several studies indeed indicate that Pds5A/B behavior on chromatin differs from that of the cohesin core. Analysis of conditional Scc1 knockout (KO) cells revealed a strong concomitant decrease in chromatin associated SMC1, SMC3, SA1 and SA2, the other core components of the cohesin complex (Ohta et al., 2016). However, chromatin levels of the cohesin associated regulatory factors Pds5A and Wapl did not decrease to similar extents and Pds5B levels remained unchanged. In line with these results, depletion of Scc1 results in dispersion, but not loss, of H3T3ph over the length of the chromosome arms (Yamagishi et al., 2010). Taken together, the data suggest that while Pds5B may bind to the cohesin complex to concentrate Pds5B at centromeres, its association with chromatin does not depend on this interaction *per se*. Intriguingly, Pds5B has been shown to directly bind to DNA via two C-terminal AT hook domains (absent in Pds5A) (Couturier et al., 2016). How these domains contribute to localization of Pds5B and Haspin during interphase and mitosis will require further investigation.

#### Regulation of Haspin activity

Apart from its localization, Haspin activity itself is subject to further regulation, adding an extra layer of complexity. Haspin is an atypical kinase and its activity is not controlled by phosphorylation of the activation loop (Eswaran et al., 2009; Villa et al., 2009). Instead, the unstructured N-terminal part of the protein harbors a unique autoinhibitory motif termed the Haspin Basic Inhibitory Segment or HBIS (Ghenoiu et al., 2013; Zhou et al., 2014). During mitosis, Cdk1, Plk1, and Aurora B phosphorylate multiple residues in the N-terminal region of Haspin, thereby releasing the HBIS and thus resulting in full Haspin activity (Wang et al., 2011; Ghenoiu et al., 2013; Zhou et al., 2014). This highlights a key positive feedback loop through which Aurora B activity contributes to its own localization.

Despite the presence of the HBIS sequence, recombinant Haspin isolated from *E. coli* or from interphase cell extracts displayed robust activity toward H3 *in vitro* (Dai et al., 2005; Ghenoiu et al., 2013). Furthermore, a Haspin mutant isolated from mitotic cells, but lacking 11 putative Aurora B consensus sites, was still able to phosphorylate H3 *in vitro*, despite displaying a strong decrease in H3T3 phosphorylation in cells (Wang et al., 2011). While a more detailed analysis has shown that phosphorylation by Plk1 modestly stimulates the kinetics of H3T3 phosphorylation by Haspin (Ghenoiu et al., 2013) the discrepancy between *in vitro* and *in vivo* kinase activity of Haspin mutants suggest that in cells the substrate H3T3 is regulated by additional factors.

#### Regulation of H3T3 phosphorylation

H3T3 phosphorylation is reversed by PP1γ. The activity of PP1 typically relies on the association with a regulatory factor that controls its targeting to its substrates. Activity toward H3T3ph requires the PP1γ-interacting protein Repo-Man, which recruits PP1γ to chromatin. Together they control H3T3ph levels through an intricate circuit that further depends on the activity of Cdk1 and Aurora B (Trinkle-Mulcahy et al., 2006; Qian et al., 2011; Vagnarelli et al., 2011). During mitosis, Cdk1-mediated phosphorylation of Repo-Man largely represses its interaction with PP1γ and chromatin (Figures 3B,C). However, low levels of chromatin bound PP1γ-Repo-Man appear sufficient for removal of H3T3ph on the chromosome arms, while other mitotic substrates remain below the threshold for dephosphorylation. Centromeric H3T3ph is protected from PP1γ-Repo-Man activity by Aurora B, which phosphorylates Repo-Man on serine 893 (S893). This results in a strong decrease in affinity for histones, thus effectively reducing PP1γ activity at centromeres (Qian et al., 2013). On the other hand, Cdk1 activity promotes the interaction between Repo-Man and PP2A, which can dephosphorylate Repo-Man S893, thereby controlling basal chromatin levels of PP1γ-Repo-Man during mitosis (Qian et al., 2015). This feedback between PP1γ/PP2A/Repo-Man and Cdk1 and Aurora B contributes to restricting H3T3ph, and thus the CPC, to centromeres. Furthermore, it facilitates the switch-like behavior observed at mitotic exit: the drop in Cdk1 activity upon anaphase onset results in full activity of PP1γ-Repo-Man on chromatin, thereby allowing rapid and complete dephosphorylation of mitotic substrates (Figure 3D).

#### Regulation of haspin at the substrate level: epigenetic context

**Figure 4:**
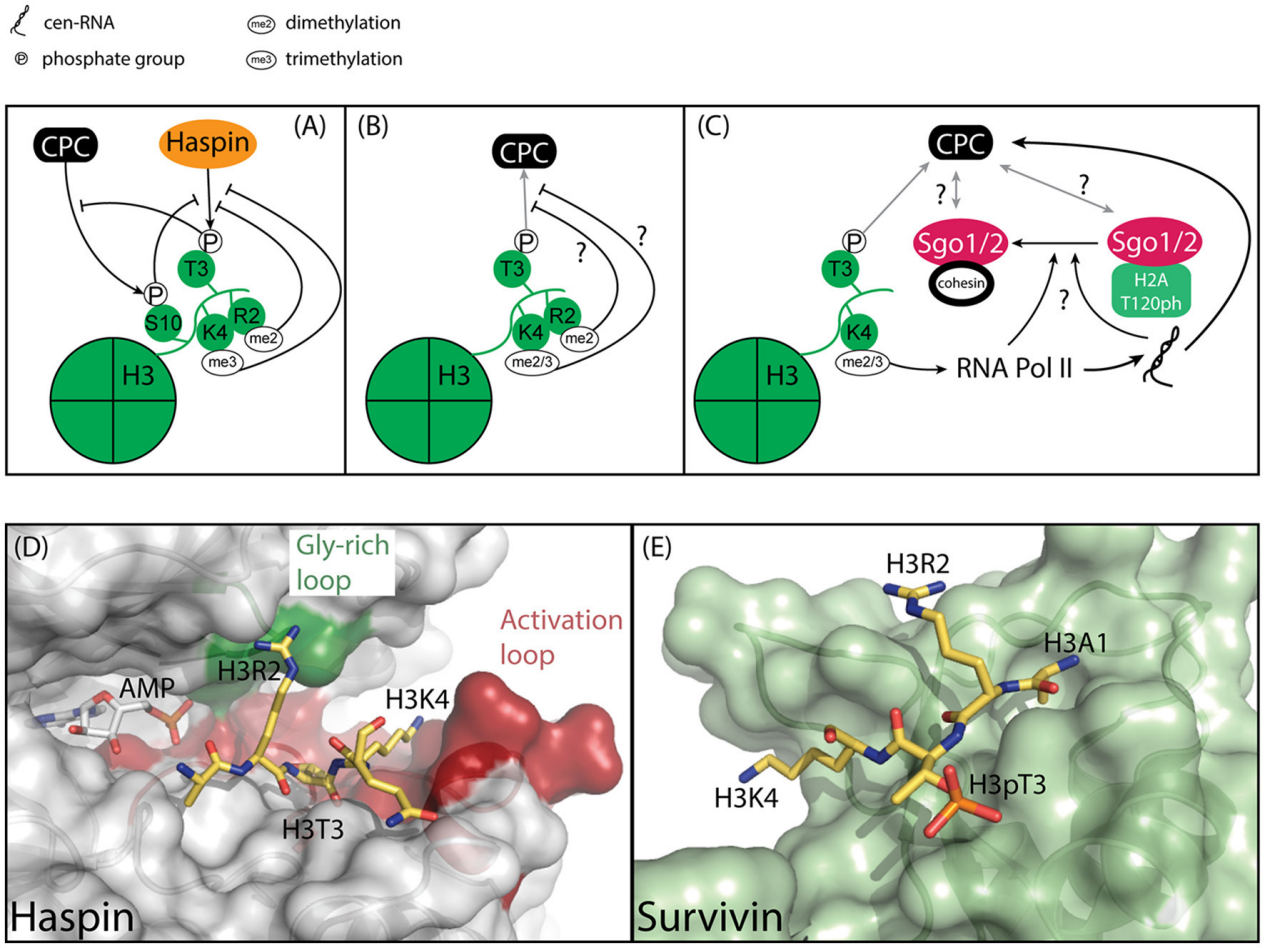
Model of how the epigenetic context of H3T3 might influence its phosphorylation by Haspin and its capacity to recruit the CPC. **(A)** Methylation and phosphorylation of the histone tail of H3 impede H3T3 phosphorylation. H3T3ph impedes phosphorylation of H3S10. **(B)** Methylation of residues adjacent to H3T3ph could hinder the interaction of H3T3ph with Survivin, thereby impeding CPC recruitment. **(C)** H3K4 di/trimethylation is associated with centromere transcription, which is required for full Aurora B activity and CPC localization to the inner centromere. Centromeric transcription and/or the resulting transcript also regulate(s) Sgo1/2 translocation from the kinetochore-proximal centromere to the inner centromere, however it is unclear if this is related to the effect of transcription on the CPC. **(D)** Close-up of the active site of Haspin (gray) bound to its substrate, Histone H3 (yellow) (PDB ID: 2WB8). The AMP moiety is modeled based on PDB ID 3DLZ. The structure reveals extensive interaction between H3R2 and the Gly-rich loop, depicted in green, and H3K4 and the activation loop, depicted in red. As such, modifications of residues adjacent to H3T3 could influence substrate binding. **(E)** Close-up of Survivin (light green), bound to a Histone H3 peptide (yellow) (PDB ID: 3UIG). The structure depicts the interactions between H3 and the BIR domain of Survivin.

Survivin is a “reader” of H3T3ph (Kelly et al., 2010; Wang et al., 2010). However, Histone H3 tails are subject to many more posttranslational modifications and their juxtaposition to H3T3 makes it tempting to speculate they could contribute to regulation of H3T3 accessibility to Haspin or to Survivin binding. In fact, crosstalk between multiple histone marks is commonly observed as a means for reversibly controlling chromatin association of proteins. For example, heterochromatin protein 1 (HP1) specifically interacts with H3 when lysine 9 is trimethylated. However, phosphorylation of H3S10 by Aurora B results in eviction of HP1, thereby creating a so-called “methyl/phosphor” switch (Fischle et al., 2005; Hirota et al., 2005). In case of H3T3, methylation of the adjacent residues H3R2 and H3K4 has been shown to negatively influence Haspin activity toward H3T3 *in vitro* (Figures 4A,D) (Eswaran et al., 2009; Villa et al., 2009; Han et al., 2011; Karimi-Ashtiyani and Houben, 2013).

This raises several questions: Do these marks occur during mitosis and if so, where? Several observations suggest this could be the case. First, despite the fact that H3K4Me_3_ suppresses H3T3 phosphorylation by Haspin *in vitro*, it has been observed in mitotic cells in a combinatorial mark together with H3T3ph and H3R8Me_2_ (Markaki et al., 2009). Moreover, this mark was highly enriched at centromeres. Unfortunately, the functional significance of this modification remains unresolved. Intriguingly, H3T3ph has been shown to decrease the binding of the transcription factor complex TFIID to H3K4Me_3_, suggesting the presence of a “methyl/phosphor” switch that represses transcription during mitosis (Varier et al., 2010). At the same time, H3K4Me_2_ dependent transcription at centromeres does occur and has been shown to play an important role in regulating centromere function, including the regulation of Aurora B activity (Figure 4C) (Sullivan and Karpen, 2004; Jambhekar et al., 2014; Blower, 2016; Molina et al., 2016; McNulty et al., 2017). This in turn raises the question if Haspin contributes to the regulation of centromeric transcription. Perhaps the confinement of Haspin and H3T3ph to the inner centromere restricts transcriptional start sites to kinetochore-proximal centromeres, suggesting the presence of multiple functional domains within centromeres (Sullivan and Karpen, 2004). If and how Haspin controls centromeric transcription will require further analysis.

Of note, while modification of residues adjacent to H3T3 clearly influences H3T3 phosphorylation, it remains unclear how modifications in the vicinity of H3T3ph would influence binding to Survivin. Analysis of the structure of a complex between Survivin and a H3 peptide reveals extensive interactions between H3R2 and H3K4 with the BIR domain of Survivin (Figures 4B,E) (Jeyaprakash et al., 2007; Kelly et al., 2010; Niedzialkowska et al., 2012). However, while both H3R2 and H3K4 make multiple electrostatic interactions with Survivin these side chains adopt an extended conformation over the surface of Survivin, suggesting ample space to accommodate additional modifications (Figures 4B,E). Ultimately, if and how H3 modification beyond H3T3 phosphorylation affect Survivin binding beyond H3T3 phosphorylation will need to be addressed experimentally.

Interestingly, H3S10 phosphorylation by Aurora B was shown to significantly impede H3T3 phosphorylation *in vitro* and vice versa, suggesting that these “common” mitotic histone marks may not coexist on the same histone tail (Han et al., 2011). This type of crosstalk again suggests the possible presence of multiple domains, each carrying unique combinatorial marks within the 3D organization of centromeres. The presence of such domains and how they may contribute to CPC localization and chromosome segregation during mitosis remain unclear and will require further analysis.

#### Bub1, H2AT120ph and the inter-kinetochore axis

Haspin activity controls CPC localization to the inter-sister chromatid region by virtue of its association with cohesin (Figures 1, 2, 3B). On the other hand, Bub1 kinase activity concentrates the inter-sister pool of the CPC at centromeres. Bub1 is thought to exert its control over CPC localization through recruitment of Sgo1 and Sgo2 but how Bub1 and Sgo1/2 collaborate to control CPC localization remains poorly understood at the molecular level.

**Figure 5:**
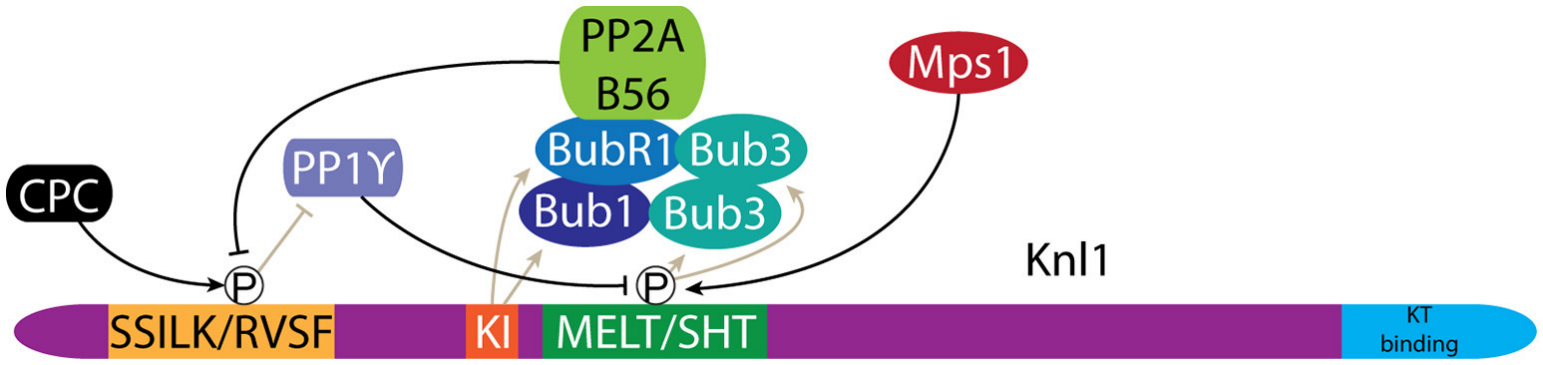
Bub1 recruitment to kinetochores is regulated by phosphorylation of Knl1. Phosphorylation of MELT/SHT motifs in Knl1 by Mps1 mediates recruitment of the Bub proteins. A negative feedback loop is created through the recruitment of PP2A/B56, which antagonizes phosphorylation of the SSILK/RVSF motifs by the CPC. Dephosphorylation of the SSILK/RVSF motifs allows PP1γ binding, which in turn antagonizes MELT/SHT phosphorylation.

Bub1 is recruited to chromatin via its association with the kinetochore protein Knl1 (Figure 5). Importantly, this restricts Bub1 activity to the centromere region (Figures 1, 2) (Kiyomitsu et al., 2007, 2011). Knl1 forms an important signaling platform within the KMN network and its location at the microtubule-kinetochore interface allows it to control mitotic checkpoint signaling and chromosome congression (Caldas and DeLuca, 2014). Bub1 is recruited to Knl1 as part of a larger complex that further consists of Bub3 and BubR1 (collectively referred to as the Bubs) (Taylor et al., 1998). Recruitment of the Bubs depends on the activity of the mitotic kinase Mps1, which phosphorylates an array of so-called MELT/SHT motifs in Knl1 (London et al., 2012; Shepperd et al., 2012; Yamagishi et al., 2012; Primorac et al., 2013; Vleugel et al., 2013, 2015). Bub3 specifically recognizes these motifs, resulting in recruitment of the Bubs to the kinetochore (London et al., 2012; Shepperd et al., 2012; Yamagishi et al., 2012; Primorac et al., 2013; Vleugel et al., 2013, 2015). The recruitment of the Bubs is further enhanced through a direct interaction between the TPR domains of Bub1 and BubR1 and two “KI” motifs in Knl1 (Bolanos-Garcia et al., 2011; Kiyomitsu et al., 2011; Krenn et al., 2012).

The level of Bub1 at kinetochores is tightly controlled through an intricate regulatory circuit that couples the microtubule attachment status of the kinetochore to Bub1 levels. Bub1 levels are higher at unattached vs. microtubule attached kinetochores (Jablonski et al., 1998; Hoffman et al., 2001; Skoufias et al., 2001; Taylor et al., 2001; Ditchfield et al., 2003), but also appears higher at attached kinetochores with reduced tension across centromeres (Skoufias et al., 2001; Taylor et al., 2001). PP1γ activity antagonizes Mps1 dependent recruitment of the Bubs (Figure 5). Knl1 directly recruits PP1γ to kinetochores where it binds to a conserved SSILK/RVSF motif. PP1γ levels are in turn controlled by the activities of Aurora B and PP2A. Aurora B directly phosphorylates the Knl1 SSILK/RVSF motif, which inhibits PP1γ binding while PP2A antagonizes Aurora B phosphorylation, thereby promoting PP1γ binding to Knl1 (Figure 5) (Liu et al., 2010; Nijenhuis et al., 2014). Interestingly, it is BubR1 that recruits PP2A to kinetochores (Suijkerbuijk et al., 2012; Kruse et al., 2013; Xu et al., 2013). As such, the association of the Bubs with Knl1 at the same time primes their removal. This negative feedback ensures a responsive mitotic checkpoint signal but likely also contributes to controlling CPC levels at centromeres (Nijenhuis et al., 2014). Indeed, both Sgo1 and Aurora B levels are higher at unattached kinetochores (Salimian et al., 2011; Liu et al., 2013a; Meppelink et al., 2015). This is in line with the role of Aurora B in establishing bi-orientation. Early in mitosis high Aurora B activity is required to destabilize potential erroneous kinetochore-microtubule (KT-MT) interactions, while Aurora B activity must later be down-regulated to support formation of stable bi-oriented KT-MT interactions (see discussion below) (Salimian et al., 2011; Krenn and Musacchio, 2015).

#### The role of Sgo1 in CPC localization

Bub1-mediated phosphorylation of H2AT120 directly recruits Sgo1 and Sgo2 to centromeres (Tang et al., 2004; Kitajima et al., 2005; Gómez et al., 2007; Kawashima et al., 2010; Tanno et al., 2010; Liu et al., 2013b). How then does this contribute to the (inner) centromere localization of the CPC? First, by recruiting Sgo1/2 to centromeres Bub1 ensures centromeres are protected from the cohesin removing activity of the prophase pathway (McGuinness et al., 2005; Kitajima et al., 2006; Kawashima et al., 2010; Tanno et al., 2010), which likely contributes to restricting cohesin associated Haspin to centromeres (see above). Depletion or inhibition of Bub1 results in so-called closed arm chromosomes, as the prophase pathway no longer removes cohesin from the chromosome arms. This effect depends on Sgo1 (Kitajima et al., 2005), which, along with Haspin and the CPC, is redistributed along the inter-sister chromatid axis (Ricke et al., 2012; Liu et al., 2013a; Baron et al., 2016).

The data so far suggest that the Bub1>H2AT120ph>Sgo1/2 pathway might simply act as a roadblock against cohesin removal, and thereby Haspin removal, from centromeres. As such this pathway would indirectly contribute to the enrichment of H3T3ph at the inner centromere. Yet, yeast-2-hybrid experiments suggest that Sgo1/2 directly interact with Borealin (Tsukahara et al., 2010; Lee et al., 2014). This implies that Sgo1/2 also play a direct role in CPC centromere localization, but the exact mechanism remains poorly understood. In fission yeast, Sgo2 is the main contributor to CPC localization during mitosis, and while Sgo2 has been shown to contribute to CPC localization in human cells its role remains understudied (Table 1) (Yamagishi et al., 2010). We will therefore limit our discussion to the role of Sgo1.

Recruitment of Sgo1 to the inner centromere is a two-step process. First, Sgo1 is recruited to H2AT120ph, located at two centromeric foci, proximal to the kinetochores (Figure 2) (Lee et al., 2008; Liu et al., 2013a, 2015). Then, in a second step, Sgo1 moves to the inner centromere by binding to the cohesin complex (Liu et al., 2013a). Sgo1 binds cohesin at the interface between the SA2 and Scc1 subunits and this interaction further requires phosphorylation of Sgo1T346 by Cdk1 (Liu et al., 2013b; Hara et al., 2014). Sgo1 mutants that are unable to bind to H2AT120ph no longer accumulate at the (inner) centromere, while mutations that prevent binding to cohesin result in accumulation of Sgo1 at the centromere pools of H2AT120ph, proximal to the kinetochores (McGuinness et al., 2005; Kawashima et al., 2010; Liu et al., 2013a,b, 2015). This suggests that the association of Sgo1 with H2AT120ph forms a crucial intermediate step prior to translocating to the inner centromere (Liu et al., 2015). Interestingly, Bub1 activity at kinetochores also contributes to the localization of actively transcribing RNA polymerase II (Pol II) (Chan et al., 2012; Liu et al., 2015). Moreover, Bub1-dependent transcription at centromeres is required for translocation of Sgo1 to the inner centromere. Sgo1 was shown to bind to RNA and Pol II, but how centromeric transcription results in inner centromere localization remains unclear (Liu et al., 2015). Centromeric transcription has been shown to generate multiple species of lncRNAs that play important roles in kinetochore assembly and regulation of CPC activity (Sullivan and Karpen, 2004; Jambhekar et al., 2014; Blower, 2016; McNulty et al., 2017). Importantly, since Aurora B activity also contributes to Sgo1 localization these studies should be interpreted cautiously as indirect effects of centromeric RNA on Sgo1 localization via Aurora B cannot be excluded (van der Waal et al., 2012; Lee et al., 2014).

How then does Sgo1 contribute to CPC localization? Sgo1 has been shown to interact with the CPC, specifically with Borealin in a Cdk1 dependent fashion (Kawashima et al., 2007; Tsukahara et al., 2010; Jeyaprakash et al., 2011; Liu et al., 2015). This makes it tempting to speculate that the CPC tags along with Sgo1 toward the inner centromere. First, Sgo1 would recruit the CPC toward H2AT120ph at the kinetochore-proximal centromere. The subsequent association of Sgo1 with cohesin may drag the CPC toward the inner centromere, aided by the interaction between Survivin and H3T3ph (Figure 2). However, this model raises several questions: if the CPC and Sgo1 translocate to the inner centromere as a single unit then H3T3ph would also be expected to contribute to inner centromere localization of Sgo1. Indeed, knockout (KO) of Haspin in human cells was shown to result in redistribution of Sgo1 from the inner centromere toward the two kinetochore-proximal pools of H2AT120ph (Zhou et al., 2017). However, these results must be interpreted cautiously since Haspin was also shown to directly contribute to protection of centromeric cohesion (Dai et al., 2006; Zhou et al., 2017). In fact, depletion of Wapl from Haspin KO cells was sufficient to restore inner centromere localization of Sgo1, suggesting that H3T3ph is not required for inner centromere localization of Sgo1. Since the localization of the CPC was not addressed in these studies (Zhou et al., 2017), it remains to be seen if depletion of Wapl in Haspin KO cells is able to rescue inner centromere localization of the CPC.

Other observations argue against the “tag along” model. First, mouse embryonic fibroblasts engineered to express kinase dead Bub1 where shown to have closed arms with Aurora B localized along the inter-sister chromatid axis (Ricke et al., 2012). This may imply that delocalized Sgo1, caused by the lack of Bub1 activity, results in redistribution of the CPC, along the lines of the “tag along” model. However, specific targeting of Sgo1 to centromeres, through ectopic expression of Sgo1 fused to the centromere-targeting domain of CENP-B, was unable to rescue (inner) centromere enrichment of the CPC (Ricke et al., 2012). Furthermore, while depletion of Sgo1 reduces centromere levels of the CPC, overexpression of Sgo1 does not result in a concomitant increase of the CPC (Meppelink et al., 2015). This is in contrast to PP2A, which binds directly to an N-terminal coiled coil in Sgo1, and whose levels at the inner centromere strongly correlate with Sgo1 (Meppelink et al., 2015). This suggests that while Sgo1 contributes to CPC localization at centromeres, the inner centromere pool of Sgo1 may not be associated with the CPC. Of note, mapping of the Borealin binding site in Sgo1 has pinpointed the N-terminal coiled coil that also binds PP2A, raising the question if the interaction between Sgo1–PP2A and Sgo1–CPC are perhaps mutually exclusive (Xu et al., 2009; Tsukahara et al., 2010). For now, the interaction between the CPC and Sgo1 remains poorly characterized. Future analysis should allow for the identification of Sgo1 mutants that specifically disrupt its interaction with the CPC, to shed light on the molecular basis of how Sgo1 contributes to the localization of the CPC.

### Aurora B-mediated control of CPC localization

It is clear that complex signaling underlies the defined localization of the CPC at the inner centromere. It is intriguing that almost every pathway that contributes to confining the CPC to the inner centromere is under control of Aurora B activity itself. This includes the important roles of Aurora B in the prophase pathway, in the maintenance of centromeric cohesion, in Haspin activation, in the control of H3T3ph levels and in the regulation of Bub1 and Sgo1 levels at the kinetochore and (inner) centromere, respectively. Furthermore, Aurora B phosphorylation of H2AXS121 at centromeres has been shown to contribute to (inner) centromere localization of the CPC (Shimada et al., 2016). As these various pathways converge to concentrate the CPC at the inner centromere they further contribute to the positive feedback cycle as clustering has been shown to contribute to full activation of Aurora B by facilitating the auto-phosphorylation, in trans, of its activation loop (Bishop and Schumacher, 2002; Kelly et al., 2007).

At the same time these data highlight an important challenge in studying the localization of the CPC. Extensive crosstalk between cohesin, Haspin, Bub1, Sgo1, and the CPC make it difficult to explain at the molecular level the phenotypic observations following perturbation of the system. Loss of Sgo1 and Haspin both result in a loss of (centromere) cohesion and thus in essence in the absence of an inner centromere. The identification of separation of function alleles that uncouple the multiple functions of these proteins will be required to further unravel the underlying signaling that controls CPC localization.

## Is inner centromere localization of the CPC required for CPC function during mitosis?

As explained above, an intricate and evolutionary conserved signaling network that is operational in our cells directs the CPC toward the inner centromere region of the duplicated chromosomes. This typical localization of the CPC has for long been considered crucial for the various mitotic functions executed by its enzymatic subunit Aurora B. However, several recent pieces of data challenge this view. Below we will summarize the contribution of the CPC to error-free chromosome segregation and discuss the various lines of evidence that argue for or against the need for precise localization of the CPC at the inner centromere for its activities contributing to faithful chromosome segregation.

### The CPC is essential to achieve chromosome bi-orientation

Error-free chromosome segregation requires that all the duplicated chromosomes become bi-oriented on the mitotic spindle. This means that the kinetochores of the sister chromatids need to become attached to microtubules derived from opposite poles of the mitotic spindle prior to anaphase onset. Through phosphorylation of outer kinetochore proteins that directly bind to spindle microtubules, such as components of the KMN network (particularly Ndc80/Hec1), Aurora B lowers their microtubule binding affinity and as such creates a dynamic KT-MT interface where individual microtubules continuously bind the kinetochore and are rapidly released. This high kinetochore microtubule (kMT) turnover helps to resolve “unwanted” non-bipolar KT-MT interactions, such as syntelic (sister-kinetochores are bound by microtubules from the same pole) and merotelic (one of the sister kinetochores is bound by microtubules from opposite spindle poles) attachments, that would otherwise give rise to mis-segregating chromosomes in anaphase, resulting in aneuploid daughter cells (Thompson et al., 2010). This process is frequently referred to as “error correction.” A consequence of kMT detachment is an unattached kinetochore that activates the mitotic checkpoint, a surveillance mechanism that prevents the onset of anaphase until all kinetochores have become stably connected to microtubules of the mitotic spindle (Musacchio, 2015). Aurora B also contributes to the mitotic checkpoint in a more direct manner by facilitating the rapid kinetochore recruitment of the essential checkpoint kinase Mps1 at the onset of mitosis (Santaguida et al., 2011; Saurin et al., 2011). This dual activity of Aurora B ensures that bi-oriented attachments can be established before anaphase onset. Obviously, kMT turnover needs to eventually diminish to allow the stabilization of bi-oriented attachments and silencing of the mitotic checkpoint. The switch from dynamic to more stable kMT interactions on bi-oriented chromosomes is accompanied by tension across and within the sister-kinetochores (Nicklas and Koch, 1969; Ault and Nicklas, 1989; Maresca and Salmon, 2009; Uchida et al., 2009). Tension is generated by opposing microtubule pulling forces that are counteracted by centromeric cohesin, which holds the sister chromatids together, as well as by the inner kinetochore proteins CENP-T, CENP-H/I/K/M, and CENP-C, which act as linkers between the core centromere protein CENP-A and the microtubule binding site of the kinetochore Ndc80/Hec1 (Nicklas and Koch, 1969; Ault and Nicklas, 1989; Suzuki et al., 2014; Musacchio and Desai, 2017). At least *in vitro*, tension itself stabilizes KT-MT attachments through a catch-bond like mechanism (Akiyoshi et al., 2010). Yet, tension also increases the distance between the sister-kinetochores, as well as between the inner centromere where Aurora B is localized, and the outer kinetochore where its MT binding substrates reside (Wan et al., 2009). This gave rise to the “spatial separation model,” which explains the stability of KT-MT attachments by the proximity of Aurora B to its kinetochore substrates: mal-attachments are destabilized because Aurora B can reach its outer kinetochore substrates and phosphorylate them, while bi-oriented (amphitelic) attachments are stabilized because the opposing microtubules pulling forces generated on bi-oriented chromosomes pull the outer kinetochore substrates out of the sphere of influence of Aurora B (Tanaka et al., 2002; Andrews et al., 2004; Liu et al., 2009). Indeed, a FRET-based biosensor for Aurora B activity is phosphorylated on bi-oriented sister chromatids when placed at the centromere, but not when it is positioned at the kinetochore (Liu et al., 2009). Similarly, the level of phosphorylation of endogenous Aurora B kinetochore substrates, such as Hec1, goes down upon microtubule attachment and the generation of tension across kinetochores (Welburn et al., 2010; DeLuca et al., 2011), while hyperstretching of the kinetochore, which occurs in cells lacking CENP-T or CENP-C, causes an even greater reduction in Hec1 phosphorylation (Suzuki et al., 2014). These data seem to be in line with the view that the distance between Aurora B and its substrate contributes to the level of phosphorylation of that substrate after bi-orientation. This is further substantiated by the observation that the central region of INCENP is a ~32 nm single alpha helix (SAH) that might stretch up to 80 nm under physiological forces (Peckham and Knight, 2009; Samejima et al., 2015). This extensible SAH connects the N-terminal centromere binding domain of INCENP with its C-terminal Aurora B binding domain, and may act as a “dog-leash” allowing Aurora B to phosphorylate its outer kinetochore substrates, while being tethered to the inner centromere (Santaguida and Musacchio, 2009; Samejima et al., 2015). In line with this idea, deletion of the SAH affected Aurora B-mediated phosphorylation of outer kinetochore substrates but not of inner centromere-proximal substrates (Samejima et al., 2015; Wheelock et al., 2017).

A key condition for this tension-based spatial separation model is the confined localization of Aurora B at the inner centromere. The model predicts that placement of Aurora B closer to the outer kinetochore would preclude stabilization of bi-oriented kMTs. By replacing the N-terminal (Survivin and Borealin binding) inner centromere-targeting domain of INCENP with the centromere-binding domain of CENP-B (CB-INCENP) or with the kinetochore protein Mis12 (Mis12-INCENP), it is possible to target Aurora B close to or at the kinetochore, respectively (Liu et al., 2009). In both cases, chromosomes initially bi-orient but are not retained in the metaphase plate. This phenotype was interpreted as ongoing destabilization of amphitelically attached kMTs by kinetochore-proximal Aurora B (Liu et al., 2009). However, it was recently shown that the microtubule binding protein Hec1 can be dephosphorylated and bi-oriented attachments can be stabilized in cells with kinetochore-proximal Aurora B if cohesin removal is prevented via depletion of Wapl (Hengeveld et al., 2017). The absence of the CEN module of the CPC, comprising the INCENP N-terminus, Survivin and Borealin, appeared to weaken centromere cohesion and to accelerate cohesion fatigue, causing sister chromatids to separate before anaphase (Daum et al., 2011; Hengeveld et al., 2017). These data suggested that inner centromere localization of Aurora B is not a prerequisite for the destabilization of erroneous KT-MT attachments nor for the stabilization of correct KT-MT attachments, in line with earlier observations in budding yeast. Here, deletion of the centromere-targeting domain of the INCENP homolog Sli15 causes the CPC to localize to the mitotic spindle and to kinetochores, instead of to the inner centromere. Even though this alternative localization would preclude spatial separation between the CPC and kinetochores upon bi-orientation, these cells did not show severe defects in chromosome segregation, suggesting that stable kMT interactions could be formed (Campbell and Desai, 2013). Of note, the relocation of Aurora B to the spindle and to kinetochores by expression of an INCENP mutant lacking its centromere-targeting domain (referred to as dCEN-INCENP) is not observed in human cells or *Xenopus laevis* egg extracts supplemented with sperm chromatin. This probably explains why dCEN-INCENP fails to rescue any Aurora B function in these experimental systems: it is most likely insufficiently clustered to activate Aurora B (Vader et al., 2006; Kelly et al., 2007; Haase et al., 2017; Hengeveld et al., 2017).

### CPC inner centromere localization, what is it good for?

**Figure 6:**
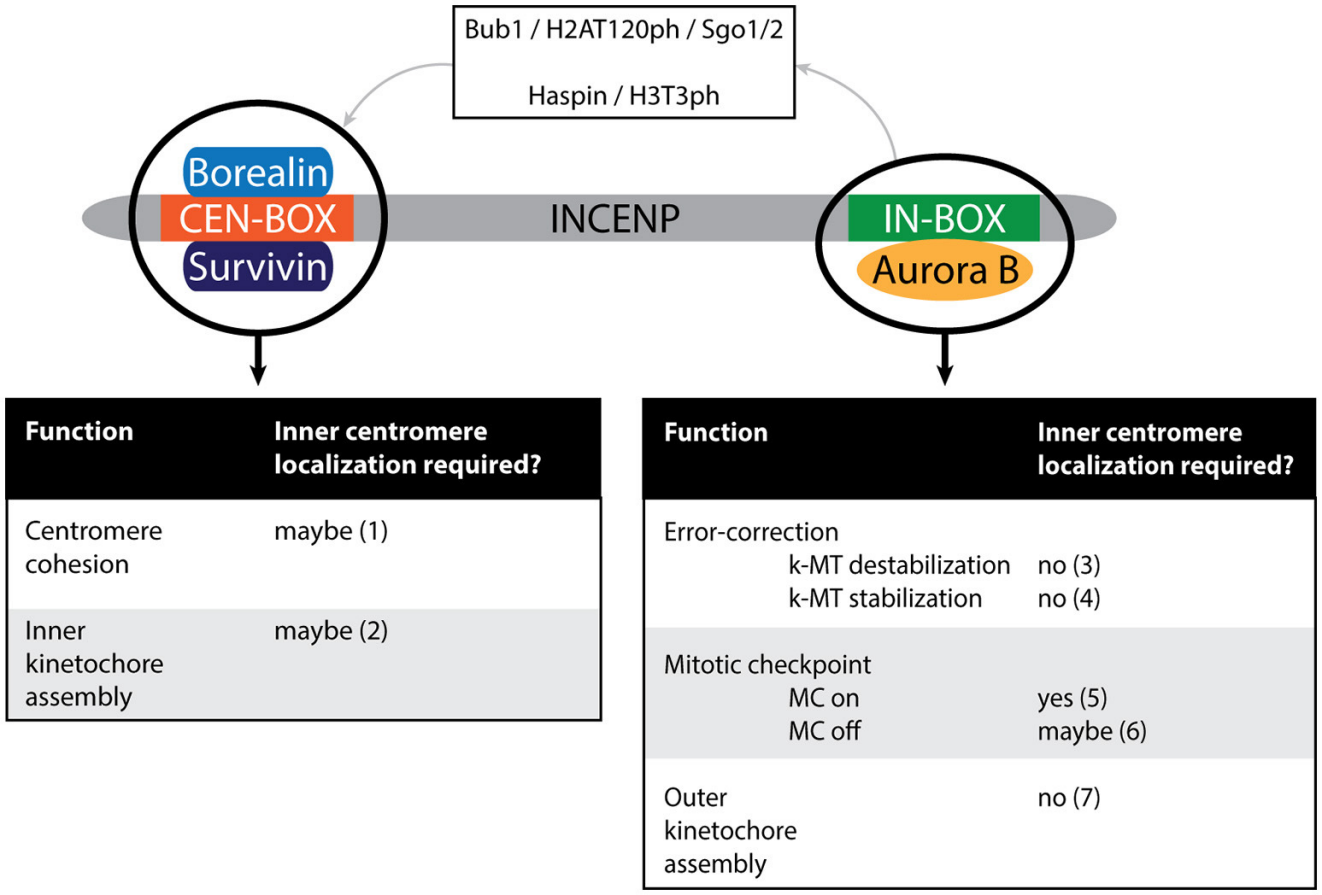
Schematic depiction of the vertebrate CPC. Different modules potentially execute different functions of the CPC: the activity module (Aurora B in conjunction with the IN-box of INCENP) or the CEN module (the CEN-box of INCENP in conjunction with Borealin and Survivin). Note that Aurora B can indirectly affect CEN module dependent functions due to the role of Aurora B in targeting the CPC to the inner centromere (as depicted by the top part of the cartoon). The dependency of each of the functions on inner centromere localization of the CPC is indicated. (1) Not tested in conditions where the CEN module is present but does not localize to the (inner) centromere (Hengeveld et al., 2017). (2) Not tested in conditions where the CEN module is present but does not localize to the (inner) centromere (Haase et al., 2017). (3) Hengeveld et al. (2017). (4) Forcing Aurora B outwards toward the kinetochore-proximal centromere (by expression of CB-INCENP) does not preclude stable KT-MT attachments in cells depleted of Wapl (to maintain cohesion), suggesting inner centromere localization does not contribute to KT-MT stabilization by spatially separating Aurora B from its kinetochore substrates (Hengeveld et al., 2017). (5) Neither CB-INCENP nor Mis12-INCENP can restore mitotic arrest in paclitaxel in the absence of endogenous INCENP (Wheelock et al., 2017). Moreover, mutation of the BIR-domain of Survivin, that prevents inner centromere localization of the CPC, causes a defect in maintaining a paclitaxel-induced arrest (Lens et al., 2006; Yue et al., 2008). (6) MC silencing is disturbed in cells expressing CB-INCENP, but it is unclear if this is due to the close proximity of the Aurora B to its kinetochore substrates or due to constitutive tethering of Aurora B to the outer centromere in this situation (Hengeveld et al., 2017). (7) Haase et al. (2017).

Based on the above, one could argue that the main function of CPC (inner) centromere localization is to cluster and activate Aurora B. If so, then all mitotic functions of the CPC should be restored when INCENP and Aurora B are clustered in an alternative manner or at an alternative location. This may be true for budding yeast, but it does not seem to be the case in other systems. In *X. laevis* egg extracts, antibody-mediated clustering of dCEN-INCENP activates Aurora B outside the chromatin. Interestingly, this unfocussed, or global Aurora B activity is sufficient to rescue outer kinetochore assembly in CPC depleted extracts, but does not support full chromosome bi-orientation (Figure 6). Interestingly, Hec1 is phosphorylated but the levels of phosphorylation are reduced on unattached kinetochores compared to wild-type (WT)-CPC extracts and remain constant after bi-orientation. Consequently, in metaphase the levels of Hec1 phosphorylation are higher in the dCEN-INCENP clustered extracts compared to WT-CPC extracts (Haase et al., 2017). Therefore, it remains unclear if the defective bi-orientation in the dCEN-INCENP clustered extracts is due to a failure in destabilizing erroneous attachments during (pro)metaphase or a failure in stabilizing bi-oriented attachments in metaphase. Addition of the CEN module to extracts with unfocussed Aurora B activity did not restore Hec1 phosphoregulation, suggesting that centromere or at least chromatin-localized Aurora B is required for Hec1 phosphoregulation in response to attachments and tension (Haase et al., 2017). As mentioned, CEN module independent clustering of Aurora B near or at kinetochores in human cells, via expression of CB-INCENP or Mis12-INCENP respectively, does not preclude the dephosphorylation of Hec1 and the formation of stabile amphitelic attachments if cohesion is stabilized (Figure 6) (Hengeveld et al., 2017). This seems to support the *X. laevis* data, suggesting that attachment and tension-dependent Hec1 phosphoregulation requires chromosome-associated Aurora B activity, but not precise inner centromere Aurora B localization. While stable amphitelic attachments can be formed in cells with kinetochore-proximal Aurora B, these cells do experience a substantial delay in metaphase because they fail to silence the mitotic checkpoint (Figure 6) (Hengeveld et al., 2017). In contrast to Hec1, the kinetochore protein Knl1 remained phosphorylated by Aurora B, thereby preventing the recruitment of PP1γ to Knl1, which is required for silencing of the mitotic checkpoint (Caldas and DeLuca, 2014; Nijenhuis et al., 2014; Hengeveld et al., 2017). It is appealing to conclude that checkpoint silencing requires the spatial separation of Aurora B and Knl1 and that this requires inner centromere localization of Aurora B. However, due to the nature of the experiments it cannot be excluded that mitotic checkpoint silencing somehow requires the removal of a potential kinetochore pool of Aurora B which is prevented in cells where Aurora B is constitutively placed near or at the kinetochore (DeLuca et al., 2011; Caldas et al., 2013).

An interesting picture emerging from these studies is that the CPC CEN module can fulfill certain CPC activities separately from the enzymatic Aurora B module, contrasting the idea that it is simply a targeting module for Aurora B (Figure 6). In human cells the CEN module strengthens centromeric cohesion to avoid premature sister chromatid separation after chromosome bi-orientation (Hengeveld et al., 2017). Moreover, in contrast to Aurora B kinase inhibition, depletion of both INCENP and Survivin from HeLa cells impairs inner kinetochore assembly; and in *X. laevis* egg extracts lacking the CPC, the CEN module suffices to rescue inner kinetochore assembly (Haase et al., 2017). It is currently unclear how the CEN module stabilizes centromeric cohesion and controls inner kinetochore assembly, but one can envision that these activities might require its presence at the inner centromere (Figure 6). In other words, maybe it is not Aurora B that needs to be at the inner centromere, but the CEN module of the CPC. If so, then perturbations that interfere with the accumulation of the CPC at the inner centromere would also affect the robustness of centromeric cohesion and/or inner kinetochore assembly. Although Haspin knockdown and KO cells experience cohesion fatigue due to weakened centromeric cohesin, this cannot be attributed solely to impaired CPC CEN module localization since Haspin itself directly controls centromeric cohesion by counteracting Wapl activity (Dai et al., 2005; Zhou et al., 2017). Evaluating centromeric cohesion and the inner kinetochore status in Survivin knockdown cells reconstituted with a Survivin BIR domain mutant that cannot bind H3T3ph (Lens et al., 2006; Yue et al., 2008; Wang et al., 2010), would be a more suitable way to test if the CEN module needs to be precisely positioned to execute these activities. Remarkably, cells expressing a Survivin BIR mutant, or with kinetochore-proximal Aurora B display a weakened mitotic checkpoint response when challenged with the microtubule stabilizing drug paclitaxel (Lens et al., 2006; Yue et al., 2008; Wheelock et al., 2017). While in the latter cells the CPC CEN module is absent, in the former cells the CEN module is present but cannot properly localize, suggesting that either an inner centromere-localized CEN module or Aurora B kinase contributes to a robust mitotic checkpoint response (Figure 6).

If inner centromere localization of the CPC is indeed necessary for chromosome bi-orientation, mitotic checkpoint signaling, centromeric cohesion protection and (inner) kinetochore assembly (Carmena et al., 2012), one would expect severe chromosome segregation defects in cells lacking either Bub1 or Haspin kinase activity, as loss of one of these activities causes a dramatic redistribution of the CPC (Tang et al., 2004; Kelly et al., 2010; Wang et al., 2010; Yamagishi et al., 2010; Ricke et al., 2012). Indeed, mouse embryonic fibroblasts (MEFs) derived from mice deficient in Bub1 kinase activity frequently enter anaphase with misaligned chromosomes, causing near-diploid aneuploidies in approximately 25% of the cells. The chromosome mis-alignment defects were rescued by expression of CB-INCENP, which relocated the majority of Aurora B to the kinetochore-proximal centromere, suggesting that the pool of Aurora B that localizes at the inter sister chromatid axis when Bub1 activity is impaired, is less efficient in error correction (Tang et al., 2004; Ricke et al., 2012). Remarkably, Haspin KO HeLa cell lines only display a moderate increase in chromosome segregation errors during unperturbed mitosis. However, when mitotic Haspin KO cells were released from a transient monopolar arrest they did display profound mitotic defects, which could be mainly explained by loss of centromeric cohesion. When cohesion was restored by depletion of Wapl, cells were delayed in establishing chromosome bi-orientation, most likely due to mislocalization of the CPC (Zhou et al., 2017). If so, it suggests that the consequences of mislocalized CPC for chromosomal stability are quite mild.

Aneuploidy is a frequent cause of embryonic lethality and a hallmark of cancer (Torres et al., 2008), however Bub1 kinase-dead mice develop normally and do not develop tumors (Ricke et al., 2012). Similarly, embryonic development in Haspin KO mice also occurs normally. However, it remains to be determined if MEFs derived from these mice experience chromosome segregation problems, and if these mice eventually develop tumors (Shimada et al., 2016). Interestingly, male Bub1 mice are subfertile and Haspin KO mice show testicular abnormalities (Ricke et al., 2012; Shimada et al., 2016). This could mean that both CPC recruitment arms and/or inner centromere localization of the CPC are needed for proper spermatocyte meiosis and fertility.

## Concluding remarks

Based on the data discussed in this review we propose that the evolutionary conservation of two CPC centromere recruitment arms ensures that sufficient amounts of active Aurora B accumulate in the vicinity of kinetochores. We suggest that it makes the KT-MT error correction system robust, thereby making dividing cells more resilient to conditions that would weaken the mitotic checkpoint or that would increase the chance of acquiring erroneous KT-MT attachments, such as disturbances in the geometry of the mitotic spindle (Ertych et al., 2014). In other words, it may safeguard chromosome segregation fidelity in anomalous situations. In addition, inner centromere localization of Aurora B may still be relevant to control the phosphorylation status of a number of outer kinetochore substrates, but not all of them. This likely also depends on the regulation of the phosphatase that dephosphorylates a particular substrate. If the antagonizing phosphatase is present at the kinetochore in relatively high amounts then even a slight change in the kinase-substrate distance may have a dramatic effect on the phosphorylation status of the substrate. Finally, the realization that the CEN module of the CPC strengthens centromere cohesion and is involved in inner kinetochore assembly opens up the possibility that the “activity” of the CEN module requires inner centromere localization.

## Author contributions

All authors listed have made a substantial, direct and intellectual contribution to the work, and approved it for publication.

### Conflict of interest statement

The authors declare that the research was conducted in the absence of any commercial or financial relationships that could be construed as a potential conflict of interest.
